# Participatory Development and Concept Testing of mHealth Messaging to Support Care Engagement and Antiretroviral Therapy Adherence for Women Living With HIV in the Southern United States: Focus Group Study

**DOI:** 10.2196/76286

**Published:** 2025-10-22

**Authors:** Sadie B Sommer, Julie V Barroso, Sarah B Bass, Marianne R Choufani, Alexander M Schoemann, Katie J Singley, Caseem C Luck, Courtney E Caiola

**Affiliations:** 1 Center for Research Development and Scholarship, School of Nursing, Vanderbilt University, 461 21st Ave. S, Nashville, TN, 37240, United States, 1 6154798547; 2 Department of Social and Behavioral Sciences, Temple University, Philadelphia, PA, United States; 3 College of Nursing, East Carolina University, Greenville, NC, United States; 4 Department of Psychology, East Carolina University, Greenville, United States

**Keywords:** mobile health, mHealth, concept testing, health messaging, HIV care engagement, antiretroviral therapy adherence, ART adherence, HIV self-management, focus groups, stigma reduction, women living with HIV

## Abstract

**Background:**

Women living with HIV in the Southern United States, or the South, face persistent and overlapping challenges to care engagement and antiretroviral therapy adherence, including HIV-related stigma, poverty, and inequitable access to health care. While mobile health (mHealth) interventions show promise for enhancing self-management and care engagement among people living with HIV, interventions tailored to women living with HIV remain limited, particularly those developed through participatory approaches that center their lived experiences.

**Objective:**

This study sought to evaluate the acceptability, comprehensibility, and personal relevance of targeted health messages developed for a proposed mHealth app tailored to women living with HIV in the South. In addition, it explored participants’ perceptions of the feasibility and desirability of the proposed intervention.

**Methods:**

This study represents phase 3 of a multistage, mixed methods project. Message content was informed by earlier phases, which included individual interviews, surveys, perceptual mapping with women living with HIV, and input from a community and clinician advisory board. In this phase, 3 focus groups (2 virtual and 1 in person) were conducted with 30 women living with HIV recruited from Southern HIV clinics and community organizations. Participants reviewed prototype wireframes and health messages, including SMS text message–style content, and provided feedback on all content. Data were analyzed using conventional content analysis.

**Results:**

Participants expressed strong interest in the proposed mHealth app and emphasized the importance of health messaging that is clear, supportive, and personally meaningful. Four key categories emerged: (1) acceptability of a tailored mHealth app, with participants noting the value of privacy, accessibility, and convenience; (2) acceptability of message content, including preferences for affirming, uplifting language and images; (3) personal relevance, particularly for messages addressing stigma, spirituality, family, and empowerment; and (4) comprehensibility, highlighting the need for plain language and visual clarity.

**Conclusions:**

These findings support the development of a tailored mHealth intervention for women living with HIV in the South. Co-designed messages that center affirmation, spirituality, and real-life challenges were perceived as acceptable, comprehensible, and highly relevant. Future work will focus on refining the content and prototype testing.

## Introduction

### Background

Despite significant advancements in HIV treatment, women living with HIV continue to face persistent barriers to sustained engagement in care and adherence to antiretroviral therapy (ART) [1], a critical strategy for achieving viral suppression [2]. This disparity is most pronounced for women living with HIV in the Southern United States, or the South, as defined by the South census region for the Centers for Disease Control and Prevention’s HIV funding [3]. Research consistently shows that women living with HIV in these states are disproportionately impacted by region-specific barriers that prevent viral suppression, such as poverty [4,5] and heightened stigma [6]. Additional geographic and structural barriers such as rurality [7], inequitable access to health care [8], and regional policies and politics [9,10] further highlight this significant and persistent health disparity. These contextual factors have contributed to higher HIV prevalence [11], low viral suppression rates [12], and increased mortality [13] for women living with HIV in the South. National data show that women living with HIV in the South are 10% to 15% less likely to achieve viral suppression than women in the Northeast and West [14], and HIV-related mortality is nearly 3 times higher in the South than in other regions [9,15]. Together, these disparities underscore the need for research that centers on the specific contexts and lived experiences of Southern women living with HIV, as well as interventions that address those needs.

### Conceptual Framework

Considering these contextual factors and layered inequities, incorporating social location, a key sensitizing concept derived from intersectional frameworks [16,17], offers a more nuanced approach to developing interventions that reflect the complexity of individual experiences. Caiola et al [16,17] offer a detailed discussion of the intersectional framework and associated concepts guiding this mixed methods study, but in short, social location refers to the dynamic consideration of contextual factors and the position that people occupy in their social hierarchies based on intersecting systems of oppression and social determinants of health. In this study, we operationalized social location to encompass both individual-level sociodemographic factors (such as race, gender, and socioeconomic status) and broader relational and structural contexts, including stigma, family and partner relationships, spirituality, and regional inequities. Acknowledging social location in message and intervention development requires moving beyond broad demographic groups such as women living with HIV or pregnant women with HIV to account for the variation in experiences and decision-making that emerge from intersecting identities and structural conditions. For example, at the individual level, women living with HIV in similar sociodemographic groups may perceive their social locations differently and make decisions about care engagement and ART adherence based on these unique interpretations.

In this study, social location served as a conceptual lens for intervention development by emphasizing how intersecting identities and structural inequities shape women’s decisions about care and adherence. This conceptual approach guided us to consider within-group differences among women living with HIV in the South and prioritize messaging that was flexible, affirming, and responsive to diverse lived experiences. Interventions are typically aimed at broad demographic groups without considering these important within-group variations and how individuals respond with vulnerability and resilience to similar experiences [18]. To most effectively reach this population, priorities must shift to first consider social location before focusing on engagement and adherence. Using this conceptual lens throughout development allows for adaptive, reflective intervention messaging that supports the whole being and well-being of women living with HIV. Achieving this requires personalized, accessible, and scalable interventions that consider all factors contributing to engagement and adherence for women living with HIV in the South [17].

### Prior Work

Digital health interventions, particularly mobile health (mHealth) apps for HIV self-management, have demonstrated positive effects on adherence, prevention, and engagement for people living with HIV [19,20]. However, these interventions have primarily been designed for the general population living with HIV, leaving a critical gap in tools that address the specific needs and contexts of women living with HIV in the South. This gap will continue to expand as the vast majority of Americans (91%) now own smartphones, including 84% of low-income Americans [21], a category inclusive of many people living with HIV [22]. mHealth has proven particularly successful for self-management across a spectrum of chronic diseases due to its ability to offer on-demand support, reminders, educational materials, and goal setting [23]. mHealth is also particularly suitable for people living with HIV, who face higher levels of stigma and discrimination, given the relative privacy and accessibility that mobile devices offer [24].

mHealth apps are increasingly being developed for specific demographics of people living with HIV [25-31] and address targeted outcomes such as stigma [32-34], stress reduction [35], resilience [36], and coping skills [37]. Some mHealth apps have been designed for specific segments of women living with HIV. For instance, Stockman et al [38] implemented a peer navigation and networking app for Black women living with HIV affected by interpersonal violence, whereas Coleman et al [39] created an SMS text messaging–based app for pregnant women living with HIV. In addition, other work has begun to explore mHealth tools for women living with HIV through pilot and proof-of-concept studies, such as the evaluation by Njie-Carr et al [40] of a technology-enhanced intervention for older women living with HIV. In these cases, women have primarily participated as recipients, reflecting progress but also underscoring the need for participatory approaches. There is a critical need for interventions that are context specific, co-designed, and grounded in women’s unique lived realities. To our knowledge, no intervention has systematically applied an intersectional framework to codevelop health messages with women living with HIV in the South. This study addresses this gap by conducting participatory concept testing of tailored mHealth messages, offering a novel approach that centers regional context, lived experience, and resilience alongside clinical adherence goals.

### Goal of This Study

This paper outlines the development process and initial concept testing of targeted HIV care engagement and medication adherence messaging and possible use of an mHealth app as the primary intervention modality for health message delivery for women living with HIV in the South. In preceding phases, we engaged women living with HIV in a structured, stepwise process to inform the development of the health messaging and the mobile app’s features, functionality, and content. This process is described in greater detail in the Phase 1, Phase 2, and Phase 3 sections, with published findings from phase 1 [1,17] and additional findings from phase 2 currently under review. Through comparative concept testing, we aimed to assess the acceptability, comprehensibility, and personal relevance of targeted health messaging and content to support medication adherence and health care engagement among women living with HIV in the South. Findings from this study will guide the development and testing of the mHealth app.

## Methods

### Design

#### Phase 1

**Figure 1.**
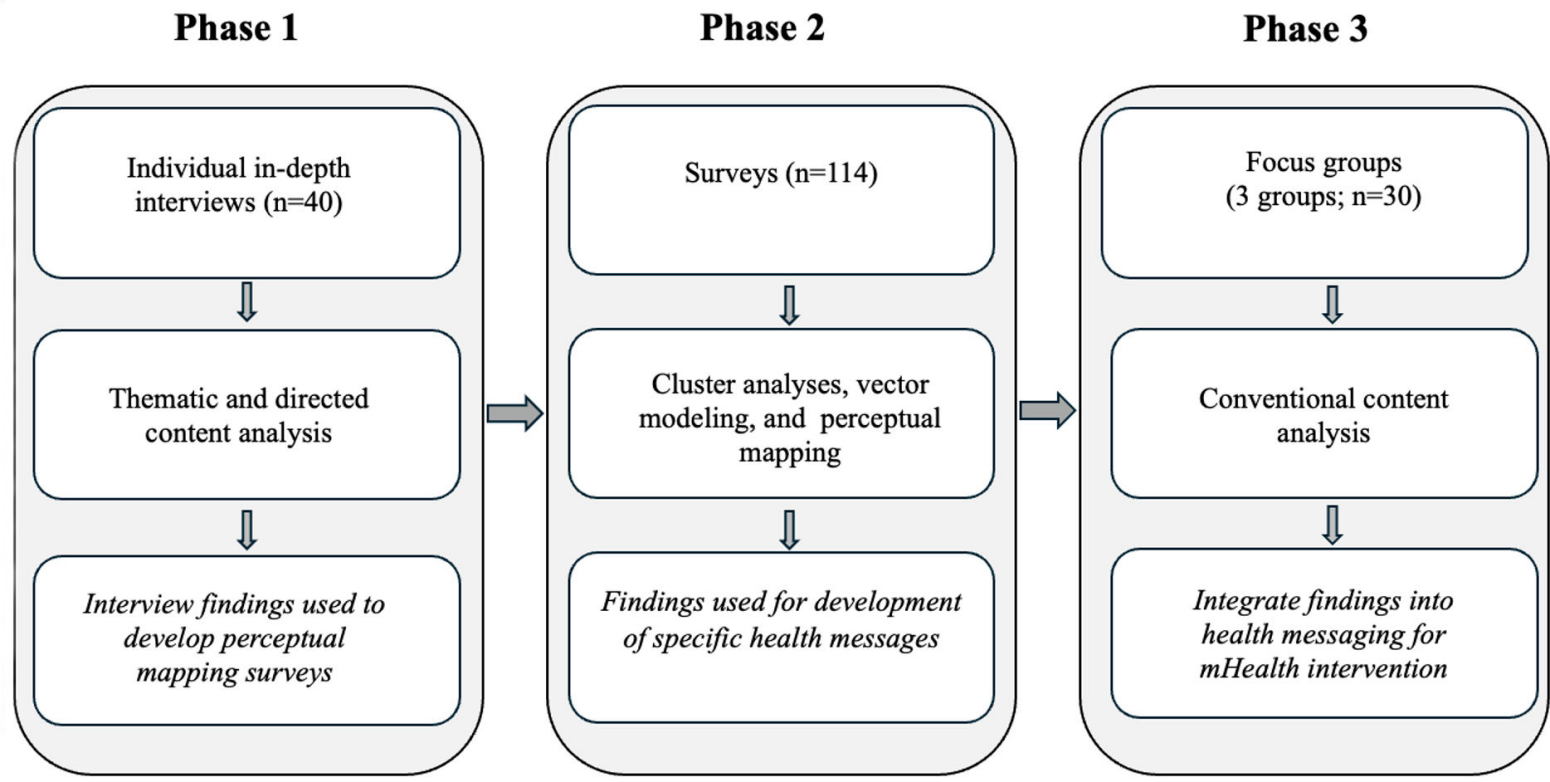
Exploratory multistage, sequential mixed methods design. mHealth: mobile health.

This paper presents the results of phase 3 of an exploratory, multistage sequential mixed methods study [41] designed to systematically develop and pretest HIV care engagement and ART medication adherence messaging (Figure 1). Data integration occurred throughout, and each phase of the work was conducted in collaboration with a community and clinician advisory board (CCAB) comprising women living with HIV and expert clinicians and researchers working with women living with HIV. Phase 1 of the sequential design included in-depth individual interviews (n=40) to determine how women living with HIV in the South perceive their social locations and make decisions regarding HIV care engagement and medication adherence. Findings from this phase have been published previously [1,17].

#### Phase 2

These findings guided the creation of surveys to further evaluate perceptions of social location and decision-making, which were administered in phase 2 (n=114). Cluster analyses, perceptual mapping, and vector modeling analyses of the phase 2 data informed the creation of a set of specific health messages designed to enhance decision-making regarding HIV care engagement and ART adherence (Table 1). Perceptual mapping and vector message modeling are novel methods that use multidimensional scaling analysis to produce mathematical 3D models, enabling the creation of highly targeted messages that conventional survey methods cannot achieve [42,43]. They also allow for comparing messaging strategies across groups by psychographic variables such as levels of stigma or by demographics to assess how persuasive messaging may be the same or different [44-46]. On the basis of vector modeling, we discerned that messaging could be incorporated to meet the needs of most women and developed messages, taglines, and content to reflect the themes identified in the analysis as most likely to be persuasive in encouraging care engagement for women living with HIV. Repetitions across clusters reflected areas of overlap identified in the data. These redundancies were intentionally retained to illustrate recurring barriers and facilitators. The findings of this work are currently under review. Perceptual mapping and vector modeling identified persuasive messages, which we refined through the intersectional framework to ensure that they reflected the women’s lived realities, including stigma, spirituality, family roles, and resource availability, and guided the categories pretested in phase 3.

**Table 1.** Messaging strategies by stigma clusters.

	HIV stigma clusters^a^			Intersectional stigma clusters^b^		
	High	Medium	Low	High	Medium	Low
Barriers to ART^c^	Too many side effects Reminds me I have HIV Don’t want friends to know	Too many side effects Get AIDS anyway Don’t want family to know Reminds me I have HIV	Inconclusive	ART is expensive Reminds me I have HIV Don’t want friends to know	ART is expensive Reminds me I have HIV Don’t want family to know	ART is expensive Don’t want family to know Don’t want friends to know
Benefits to ART	Feel more in charge Feel more confident Role model for others	Can take care of family Feel closer to partner Avoid getting sick	Inconclusive	Avoid getting sick Have a healthy, normal life	Enjoy sex more ART is safe and effective	Have a healthy, normal life Stay undetectable
Beliefs about living with HIV	Limit who knows Easy access to other resources Dating discrimination	Limit who knows	Limit who knows Discriminated against, race	Inconclusive	Limit who knows Had negative reactions to status	Inconclusive
Facilitators to ART	One pill a day Having resources and stability Having kids and family to motivate me	Having resources and stability	Inconclusive	Having resources and stability	Inconclusive	Having resources and stability Having kids and family to motivate me
Care engagement facilitators	Not great but appointment reminders help	Attending appointments prioritizes health	Appointment reminders help Gas cards and/or transportation services help	Inconclusive	Not great – Family support helps	Transportation helps Good relationship with doctor helps
Appointment barriers	Inconclusive	Inconclusive	Inconclusive	Inconclusive	Responsibilities	Inconclusive

a HIV-related stigma clusters represent messaging strategies targeting stigma specifically associated with HIV status (eg, disclosure concerns, internalized stigma, and fear of treatment).

b Intersectional stigma clusters represent messaging strategies addressing the compounding of HIV-related stigma with other stigmas tied to gender, race, socioeconomic status, or structural inequities. Both were derived from phase 2 perceptual mapping and vector modeling analyses.

c ART: antiretroviral therapy.

#### Phase 3

In phase 3, we used a descriptive qualitative design that included 3 focus groups with women living with HIV in the South, engaging a total of 30 participants. We pretested specific health messaging for comprehensibility, acceptability, and personal relevance, along with visual wireframes of how an mHealth app would use those messages and the potential of an mHealth app as the primary intervention modality for health message delivery. Recent studies confirm that focus groups are well suited for developing and tailoring mHealth interventions [47,48]. We also chose this method because it enabled efficient testing of multiple message variations and allowed for collective refinement.

To summarize the 3 phases, 40 women completed individual interviews to identify decision-making factors (phase 1), 114 women completed surveys using perceptual mapping and vector modeling to refine messaging strategies (phase 2), and 30 women participated in focus groups to assess feasibility and acceptability (phase 3) within the sequential mixed methods design.

### Ethical Considerations

The East Carolina University and Medical Center Institutional Review Board (21-001403) and the Vanderbilt University Institutional Review Board (211919) reviewed and approved all study protocols. The informed consent process was conducted by trained study personnel before all focus groups. The virtual focus group was conducted via a HIPAA (Health Insurance Portability and Accountability Act)-compliant virtual platform, Webex (Webex by Cisco). The virtual focus group participants reviewed, signed, and received a copy of the consent form electronically via a secure platform called Docusign (Docusign, Inc), and the face-to-face participants reviewed, signed, and received a paper copy. To address issues related to confidentiality, participants were asked to designate a pseudonym to use for all communications following the initial contact phone call, and each participant was assigned a unique ID number. Each participant received a $50 gift card for their time and contribution.

### Recruitment and Inclusion Criteria

Participants were recruited across a broad geographic area of the South [3], with support from CCAB members and staff at North Carolina and Tennessee recruitment sites. Recruitment strategies included (1) distributing flyers in community-based organizations and clinics to encourage self-referral; (2) obtaining referrals from health care providers for women living with HIV both in and out of care; (3) snowball sampling; and (4) sharing flyers through social media platforms and HIV and AIDS support organization email listserves connected to CCAB members, study personnel, and recruitment site staff. The eligibility criteria for participation included (1) self-identifying as a woman; (2) self-identifying as Black, Latina, or White; (3) living with HIV or AIDS; (4) being aged 18 years or older; (5) being able to read and communicate in English; and (6) being mentally competent to provide informed consent.

### Data Collection

**Figure 2.**
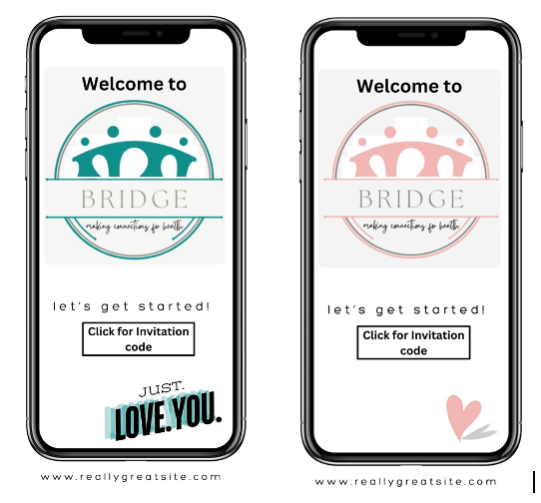
Potential mobile app initial log-in page.

To reach women living with HIV from a broad geographic distribution in the South and increase accessibility and feasibility, we conducted 2 smaller virtual focus groups (n=7 and n=5 participants) and 1 larger in-person focus group (n=18 participants). To ensure fidelity in the data collection procedures across groups and modalities (virtual vs face-to-face), the research team developed a detailed script for reviewing all the health messaging materials. The focus group interview guide included questions derived from the National Cancer Institute’s *Making Health Communication Programs Work* guide [49] to assess the comprehensibility, acceptability, and personal relevance of each specific health message. In addition, participants were asked questions regarding the acceptability of using a mobile app as the primary intervention modality for delivering health messaging. Before the focus groups, the CCAB vetted the proposed messaging and visual wireframes, offering both oral and written feedback used in refining the materials. The focus group facilitator provided a booklet containing the proposed health messaging and images to each study participant. Virtual focus group participants received an electronic copy of the booklet via their preferred email address before the focus group, whereas in-person focus group participants received a hard copy of the booklet at the focus group session. Participants were presented with variations of logos, log-in pages, potential messaging, and taglines as they would appear on their cellphones. They were also provided with examples of 12 weeks of SMS text messages to be delivered at a frequency of 3 per week. The BRIDGE Collective, or BRIDGE for short, was the proposed name of the mHealth app. Examples of weekly SMS text messages are provided in Table 2. After reviewing each option sequentially, participants were asked a series of questions about comprehensibility, acceptability, and personal relevance (eg, “Do the messages/images make sense?” “Do you like them?” “Do they speak to your personal experiences? If not, how would you suggest we change them?”). Examples of the images shown to participants are shown in Figures2^4^.

**Table 2.** Potential SMS text messages.

Category	Example message	Purpose
Reminder	“Did u take ur medication today?”	Encourages medication adherence.
Reminder	“Remember...set your alarm, take your meds every day. Text if u have ??s”	Encourages medication adherence at a specific time.
Encouragement	“Whoa, week three. U got this...text if U have ??s”	Boosts morale and promotes self-confidence.
Encouragement	“Week six! Great job doing what’s right for u! Love, the BRIDGE Collective”	Provides positive reinforcement to continue engagement.
Self-care	“When you are well, your family is well.”	Emphasizes the link between self-care and family well-being.
Self-care	“You are a role model for others when you take your medications.”	Highlights self-care as a positive example for others.
Support	“Hey, the BRIDGE Collective here. Are things getting you down? Come talk to us for help.”	Offers emotional support and connection to resources.
Support	“Remember to find your people/engage your higher power. We are here for you!”	Reinforces social and spiritual support networks.
Prompt	“Hey, the BRIDGE Collective here. Remember, if you are not working on HIV, you need to know it is working on you. Let us know if we can help!”	Open-ended prompt to check in and offer assistance.
Prompt	“See stigma for what it is: ignorance. Take Charge. The BRIDGE Collective is here for you.”	Promotes action against stigma and encourages empowerment.

**Figure 3.**
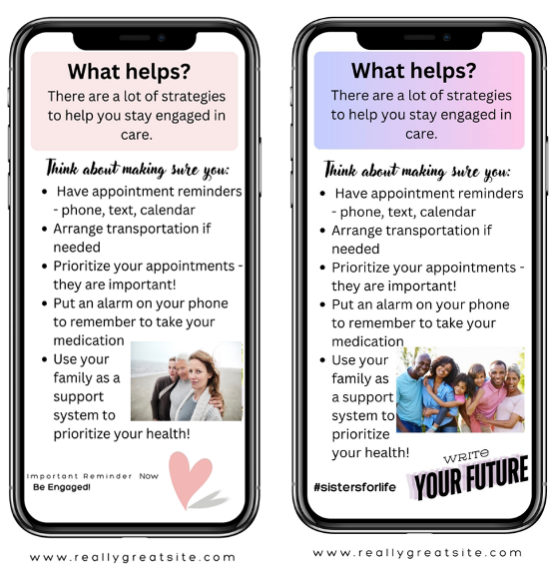
Potential messages to enhance care engagement.

**Figure 4.**
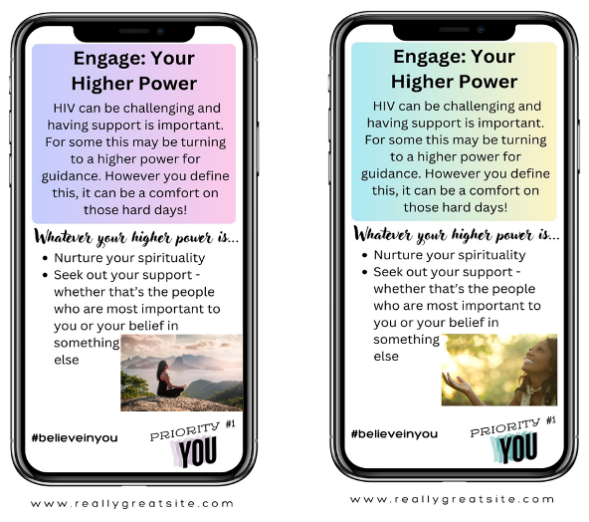
Potential spiritual messages.

### Data Management and Analysis

The sociodemographic data collected from participants in the face-to-face focus group via the paper survey were manually input into REDCap by study personnel. The sociodemographic data from all 3 focus groups were then analyzed using descriptive statistics. Audio recordings of each focus group were transcribed verbatim, verified for accuracy by study personnel, and stored on a secure drive. The qualitative data analytic team used conventional content analysis developed by Hsieh and Shannon [50] to characterize the participants’ perceptions. This approach involves inductively deriving codes from the data without predetermined categories, making it well suited to our aim of capturing participant perspectives on messaging and app features in their own words. Team members (SS and CC) read the transcripts multiple times, made note of their initial impressions, and developed initial codes derived directly from the text. To ensure trustworthiness, the team engaged in reflexivity throughout the analytic process by avoiding paraphrasing, cross-checking interpretations, and explicitly acknowledging our own positionality and potential biases during team discussions. Iterative team-based coding served as an intercoder reliability check, with discrepancies discussed until a consensus was reached [51]. The initial codes were then grouped into meaningful clusters and sorted into categories and subcategories. The field notes from each of the focus groups were also combined and integrated into the data analysis as they were used to triangulate initial findings and refine the categories and subcategories. Finally, example quotes from the data were linked with each of the proposed categories and subcategories and presented to the larger research team for reflection, discussion, and additional refinement. Analytic saturation was determined once no new themes emerged across transcripts, consistent with established standards for qualitative analysis [52].

## Results

### Sample Characteristics

A total of 30 individuals who identified as women participated in the study. The mean age of the participating women was 58 years and ranged from 29 to 75 years. Most (26/30, 87%) identified as Black, with smaller proportions identifying as White (3/30, 10%) or Latina or Hispanic (1/30, 3%). Over half (19/30, 63%) were above the federal poverty level, and most lived in nonrural county-level designations (27/30, 90%) and were currently engaged in care (27/30, 90%). The demographic characteristics of the study participants are shown in Table 3.

**Table 3.** Demographic characteristics of the participants (N=30).

Variable	Values
Age (years), mean (range)	58 (29-75)
Race or ethnicity, n (%)	
Black	26 (87)
Hispanic or Latina	3 (10)
White	1 (3)
Gender identity, n (%)	
Woman	30 (100)
Man	0 (0)
Socioeconomic status^a^, n (%)	
Above FPL^b^	19 (63)
Below FPL	11 (37)
Geographic location^c^, n (%)	
Nonrural	27 (90)
Rural	3 (10)
Engaged in HIV care, n (%)^d^	
Engaged	27 (90)
Not engaged	2 (7)
No answer	1 (3)

a Socioeconomic status was measured using the US Census Bureau standards, self-reported annual household income from all sources, and the number of people in the household [53].

b FPL: federal poverty level.

c Geographic location was measured using the participants’ self-reported state and county as the criteria and the Federal Office of Rural Health Policy designation as rural or nonrural [54].

d HIV care visit with viral load tested in the previous 6 months.

### Content Analysis

Through comprehensive analysis of the focus group transcripts and field notes, four primary categories were identified: (1) acceptability of mHealth app features and functionality, (2) acceptability of message content, (3) personal relevance of messaging, and (4) intersectional stigma and contextual barriers. These themes provide an overview of participants’ perspectives and are described in detail in the following sections.

#### Category 1: Acceptability of mHealth

##### Convenience and Accessibility

Participants expressed enthusiasm for the concept of an mHealth app tailored for women living with HIV, particularly for the convenience and flexibility it may provide. One participant stated the following:

For me, it would be very convenient. Didn’t have to get in front of a computer or show up in person.

[Online 2]

Flexibility to engage on their own time was also noted as a benefit, as one participant explained:

Well, I think an app is a good possibility because it really might not lock you down to a certain time that you had to be somewhere to communicate and associate with someone.

[Online 1]

Participants also found the idea of a free app appealing, particularly if it could help manage their health.

##### Privacy and Safety Concerns

Privacy and confidentiality were top priorities for participants. They shared concerns about protecting their HIV status if using shared phones or accessing the app in public spaces. One participant stressed the following:

It is very important because you don’t want everybody in your business. Nobody needs to know about your status unless you tell them other than you and your doctor.

[Online 2]

Another participant noted the importance of security features:

I think only women with HIV should get the invitation code because we want to be in a safe space because not everybody that would, if it was just open to anybody, then people would just know people’s business. And some people are not comfortable.

[Online 1]

In addition, adherence to privacy regulations was emphasized:

Any app or anything that we utilize, we have to be assured of confidentiality. There’s HIPAA rules, there’s everything.

[Online 1]

### Category 2: Acceptability of Message Content

#### Education and Resources

Participants saw the app as a valuable tool for providing education and resources, including information on medications, side effects, and community support. One participant noted the following:

I like that part too about having resources because a lot of times people miss out on stuff because they’re not aware of the resources available to them.

[Online 1]

Another participant suggested including information on medication, especially as a way to support women in rural areas:

Also have something about the medications as well. What are the side effects? Can you take it with blood pressure medicine? Is there an interaction with it? And I think all that because there are people that are in the rural areas that may not be able to physically get to it.

[Online 1]

#### Customizable Features

Participants expressed a desire for customization options, including the ability to adjust notification frequency and tailor content to individual needs. One participant envisioned how “folks can add themselves to each forum so that they can get notified about that forum, and then the ones that they don’t want to get notified about, they don’t” (Online 1). Translation features were also suggested:

I would like something like where you could translate Spanish. I would like to be able to have conversations with someone who...English is not their first language and be able to communicate with them just as well as I can communicate with everybody else.

[Online 1]

### Category 3: Personal Relevance of Messaging

#### Inspirational Positive Affirmations and Supportive Messaging

Participants appreciated messages that provided encouragement and positivity. One participant shared the following:

I am a big person on positive affirmations...they can help you get through some very challenging times.

[In person]

Participants appreciated messages promoting self-love, such as “Just love you” and “Priority one is you”—all language drawn directly from the qualitative data and the language used by participants in phase 1 of the study. Another participant explained how these messages resonated with her, stating that they were “something you can repeat to yourself like you standing in the mirror or not even in the mirror, but something that you can repeat to yourself to encourage yourself” (Online 2).

#### Spiritual Messages

The incorporation of spiritual messages was seen as a valuable component to many women. One participant stated the following:

Having a higher power is what really got through when I was first newly diagnosed.

[Online 1]

Other participants were receptive to messages that nurtured spirituality provided that religious language was inclusive and respectful. One participant remarked the following:

It could be the ground that is your god or that tree. It’s whatever you can call your higher power, whatever you choose it to be.

[Online 2]

#### Stigma-Related Messaging

Messages about overcoming stigma resonated with participants, emphasizing the importance of addressing discrimination from health care providers and others. One participant noted the following:

I think at one point all of us have incurred some type of judgment or stigma, so I think it’s important especially for those who are battling, still not telling people or...I don’t know, coming out the closet or however you want to say it or don’t want people to know.

[Online 2]

Another participant shared how these messages addressing HIV-related stigma could counteract difficult times:

If I’m finna give up or stop taking my medication and I kept seeing these messages pop up...it would have me think it’s more important to continue my care for me than to give up on myself.

[In person]

### Category 4: Comprehensibility of Messaging

#### Clarity of Language

There was a focus on using clear, easy-to-understand language. Participants advised against using words such as “undetectable” or “ART” without explanation. A participant explained the following:

Everyone doesn’t know all of the words. Everyone doesn’t know, you know, new people...I mean, I don’t think we should assume that people know.

[Online 2]

Participants also suggested hotlinks for complex words, ensuring that everyone, especially newly diagnosed individuals, understood the content. A participant explained the following:

I guess for me what this looks like, there should be links connected to this where they can go to find out information or more about it. [Online 1]

Another stated the following:

So they could click on it and maybe get a definition or understand what that mean.

[Online 1]

#### Visual Preferences and Layout

Feedback on the health messaging and visual designs included suggestions for larger font sizes and shorter messages. One participant suggested the following:

...as far as the font, maybe a bit small, maybe a little bit too wordy.

[Online 1]

Another remarked the following:

Yeah, I see it. It’s kind of small. It could be a little bit bigger.

[Online 2]

## Discussion

### Principal Results

This study identified 4 primary categories shaping women’s perspectives on the development of an mHealth intervention to support HIV care engagement and adherence. First, participants emphasized the acceptability of the app’s features and functionality, noting the importance of privacy, accessibility, and customizable options. Second, they highlighted the acceptability of message content, expressing strong preferences for affirming and uplifting language and images that conveyed resilience. Third, women underscored the personal relevance of messaging, stressing the value of content that felt tailored to their lived experiences and circumstances, including the importance of spirituality and faith as sources of strength and motivation. Finally, participants described the influence of intersectional stigma and contextual barriers, including structural inequities and compounded stigma, on their engagement with care and technology. Together, these findings provide a comprehensive framework for understanding how mHealth interventions can be both acceptable and meaningful for women living with HIV in the South.

We now turn to themes that warrant deeper consideration, with message comprehensibility emerging as a central point. Women consistently expressed a preference for clear, simple messaging, a finding that aligns with current National Institutes of Health recommendations for effective health communication [55], as well as SMS text messaging–based interventions for improved HIV clinical outcomes [39] and adherence [26]. Condensing findings from multistage participatory research into concise statements is a challenging but essential process to balance clarity and brevity. This refinement process ensures that only the most essential information is conveyed without unnecessary wording or visuals. This preference for succinct messaging is also supported in contemporary research that notes a shift toward shorter messaging and video content [56]. In addition, participants’ suggestions to define or hyperlink medical terminology were valuable, reinforcing best practices in digital health literacy, which emphasize the importance of accessible explanations of medical terms to enhance engagement and adherence [57]. Participants also suggested language translation, which will be a priority in future phases of development.

Privacy and security concerns also emerged as a critical consideration, with participants stressing the importance of confidentiality in protecting their HIV status. This emphasis echoes research identifying confidentiality as a primary barrier to mHealth adoption among individuals with stigmatized health conditions [58]. Similarly, Mulawa et al [24] note that, while mHealth tools may provide private avenues for engagement, ongoing fears of unintentional disclosure can hinder uptake, underscoring the importance of embedding strong security and confidentiality safeguards into intervention design. Interpreted through social location, women’s concerns about privacy reflect how intersecting experiences of HIV stigma, racism, and gendered inequities in the South heighten the risks of disclosure, making confidentiality protections central to engagement. Some participants envisioned the capacity for linking directly to individuals or group formats through social media. Green [59] found that people living with HIV were resistant to health messaging delivered through social media, viewing it as a platform that perpetuates offline inequities and promotes content that does not reflect participants’ experiences, underscoring the need to incorporate users in all stages of development. Due to the constraints of the technology, stated privacy concerns, and current literature, we are hesitant to suggest linked social media platforms as a feature for this app.

Another notable preference was for uplifting, positive messages and images, highlighting the critical role of emotional support for consistent engagement and adherence [60]. Interpreted through the lens of social location, the desire for affirming content illustrates how women living with HIV draw on encouragement as a counterbalance to the layered stigma and inequities shaping their daily lives. While practical features such as reminders, education, and resources are essential for successful care engagement and medication adherence, the women’s strong desire for encouragement highlights the importance of positive affirmations in fostering resilience and sustained engagement. This preference also aligns with research emphasizing the role of intrinsic motivation beyond scheduled reminders to sustain viral suppression [61]. In addition, focus group participants expressed interest in the ability to tailor the frequency of motivational SMS text messages to receive additional supportive content on more challenging days. For example, Schnall et al [20] showed that a self-management app with tailored features improved engagement and adherence, whereas Dworkin et al [29] demonstrated that personalized, conversational support delivered through a mobile intervention was both feasible and motivating. Together, these findings reinforce broader reviews that show that tailored notifications increase long-term retention [62]. This customization may be a key factor for sustained engagement.

The unique emphasis on spirituality in the messaging, derived from previous stages of analysis, highlights an area where existing mHealth research remains limited. Some studies have begun to highlight its potential relevance, showing, for example, that incorporating spirituality into self-management tools increases adoption and engagement [40,63]. More recently, Reid et al [64] documented that religious and spiritual coping remains a central support for Black women living with HIV, particularly older women in Southern contexts. However, reviews of HIV mHealth interventions continue to emphasize that spirituality remains insufficiently integrated [65]. Through the lens of social location, the centrality of spirituality reflects how regional culture; faith traditions; and the intersection of race, gender, and HIV status shape coping strategies and inform preferences for mHealth design. Our findings extend this work by demonstrating that, for women living with HIV in the South, spirituality is not peripheral but a central motivator for engagement, underscoring the importance of regional and psychographic targeting in designing effective interventions.

### Limitations

This study has several limitations. Although more than sufficient for concept testing, the sample size was relatively small and may not be fully representative of the broader population of women living with HIV in the region. Despite efforts to recruit women with diverse life experiences, women who are disengaged from care were not represented; their inclusion remains an essential priority for future research. There is also potential for selection bias as participants may differ systematically from those who did not enroll, particularly in their comfort with technology or engagement in care. Limited funding prevented translation in the data collection process, and the researchers were unable to recruit Hispanic or Latina women who speak Spanish as a primary language, excluding a critical demographic from this study [66]. Correspondingly, due to the specific demographics of this study, findings are not generalizable to all people living with HIV. As this study relied on focus groups to elicit perceptions of a proposed app, the findings speak to anticipated acceptability rather than demonstrated use and, thus, cannot address long-term engagement or sustained outcomes. Long-term effectiveness in improving adherence and health outcomes will require further longitudinal investigation. In addition, although an mHealth app is meant to be accessible, digital literacy, the aforementioned translation barriers, and access to reliable internet services remain potential barriers for some users, warranting further exploration of strategies to improve accessibility. Finally, because participants discussed their anticipated responses to a proposed app rather than reflecting on actual use, the findings may not accurately capture how women would engage with the technology in practice.

### Conclusions

In conclusion, this study provides robust preliminary findings to support the development of an mHealth intervention tailored to the unique needs of women living with HIV in the South. Participants emphasized the importance of engaging app features, privacy protections, and customizable options; the personal relevance of uplifting and affirming content; and the comprehensibility of clear, concise messaging. This study also identified a gap in the integration of spirituality into mHealth messaging, suggesting a potential area for future exploration. The participatory approach used in this research ensures that the proposed app reflects the lived experiences and preferences of its intended users. Future work should focus on expanding accessibility, addressing language barriers, and evaluating long-term adherence outcomes to maximize the app’s impact on health care engagement and viral suppression among women living with HIV.

## Abbreviations

ART: antiretroviral therapy
CCAB: community and clinician advisory board
HIPAA: Health Insurance Portability and Accountability Act
mHealth: mobile health
REDCap: Research Electronic Data Capture
